# Impact of gender on the survival of patients with glioblastoma

**DOI:** 10.1042/BSR20180752

**Published:** 2018-11-07

**Authors:** Minjie Tian, Wenying Ma, Yueqiu Chen, Yue Yu, Donglin Zhu, Jingping Shi, Yingdong Zhang

**Affiliations:** 1 Department of Neurology, The Affiliated Brain Hospital of Nanjing Medical University, Nanjing, Jiangsu Province, P.R. China; 2 Department of Neurology, The Affiliated Nanjing Hospital of Nanjing Medical University, Nanjing, Jiangsu Province, P.R. China

**Keywords:** gender, glioblastoma, survival, SEER

## Abstract

**Background:** Preclinical models have suggested a role for sex hormones in the development of glioblastoma multiforme (GBM). However, the impact of gender on the survival time of patients with GBM has not been fully understood. The objective of the present study was to clarify the association between gender and survival of patients with GBM by analyzing population-based data.

**Methods:** We searched the Surveillance, Epidemiology, and End-Results database who were diagnosed with GBM between 2000 and 2008 and were treated with surgery. Five-year cancer specific survival data were obtained. Kaplan–Meier methods and multivariable Cox regression models were used to analyze long-term survival outcomes and risk factors.

**Results:** A total of 6586 patients were identified; 61.5% were men and 38.5% were women. The 5-year cancer-specific survival (CSS) rates in the male and female groups were 6.8% and 8.3%, respectively (*P*=0.002 by univariate and *P*<0.001 by multivariate analysis). A stratified analysis showed that male patients always had the lowest CSS rate across localized cancer stage and different age subgroups.

**Conclusions:** Gender has prognostic value for determining GBM risk. The role of sex hormones in the development of GBM warrants further investigation.

## Background

Glioblastoma, also known as glioblastoma multiforme (GBM), is the most common primary brain tumor, with aggressive clinical manifestation [1]. The incidence rate of central nervous system tumors was reported to be 6.7 per 100,000 persons in 2000 [2]. Some researchers have demonstrated an increase in the incidence of brain tumors, which was partly result of the developments in diagnosis and changes in the classification system [3]. Despite radiotherapy plus temozolomide (TMZ) provided 2- and 5-year survival rates of 27 and 10%, median survival in GBM is generally less than 1 year, and even the patients with favorable situations, the survival month is still less than 2 years [4–7]. Except for Turcot’s syndrome and Li–Fraumeni syndrome, most GBM patients originate in a sporadic fashion without any known predisposing factors [8]. Therefore, little is known about the risk factors for brain tumors [9]. A better understanding of the distribution of GBM may provide indications of etiologic factors and contribute to the search for improved therapies.

Gender-related discrepancies in the incidence and survival of hepatocellular carcinoma [10], colorectal [11], and gastric cancers [12] have previously been reported. Above results support the protective role of estrogen in these malignancies. However, the protective role in GBM has not been investigated in a large population. To further clarify the issue of gender on GBM prognosis, Surveillance, Epidemiology, and End Results (SEER) population-based data were analyzed in our study.

## Methods

### Patients

The current SEER database consists of 17 population-based cancer registries representing approximately 26% of the U.S. population. The SEER Cancer Statistics Review (http://seer.cancer.gov/data/citation.html)—a report on the most recent cancer incidence, mortality, survival, prevalence, and lifetime risk statistics—is published annually by the Data Analysis and Interpretation Branch of the National Cancer Institute (Rockville, MD, U.S.A.). SEER data contain no identifiers and are publicly available for studies of cancer-based epidemiology and survival analysis. The National Cancer Institute’s SEER*Stat software, version 8.1.5 (Surveillance Research Program; www.seer.cancer.gov/seerstat) was used to identify patients whose pathological diagnosis as glioblastoma based on International Classification of Diseases for Oncology (ICD-O) topography codes (C71.0–C71.9) between 2000 and 2008. The definition of anatomical primary site of brain tumors was restricted to the following: C71.0-Cerebrum, C71.1-Frontal lobe, C71.2-Temporal lobe, C71.3-Parietal lobe, C71.4-Occipital lobe, C71.5-Ventricle, C71.6-Cerebellum, C71.7-Brain stem, C71.8-Overlapping lesion of brain, C71.9-Brain, and brain sites not otherwise specified (NOS). Morphology codes for glioblastoma were expanded to include the following histologies: 9440, 9441 and 9442 (i.e. glioblastoma, NOS, Giant cell glioblastoma, and Gliosarcoma). Only patients who underwent surgical treatment and who were between 18 and 70 years old at the time of diagnosis were included. Patients were excluded if they had incomplete staging, distant metastasis, or lacked an evaluation of histological type or follow-up. Age, sex, race, histological type, stage, tumor grade and size, and cancer-specific survival (CSS) were assessed. Adjuvant chemotherapy was not evaluated, since the SEER registry does not have this information. Tumor-node-metastasis classification was restaged according to criteria described in the American Joint Committee on Cancer Staging Manual (7th edition, 2010). The primary endpoint of the present study was CSS, which was calculated from the date of diagnosis to the date of cancer-related death. Deaths attributable to cancer were treated as events and deaths from other causes were treated as censored observations.

### Ethics statement

The present study was based on public data from the SEER database, and permission was obtained to access the files (reference no. 12578-Nov2013). The analysis did not involve interaction with human subjects or use personal identifying information. The study did not require informed consent and was approved by the Review Board of Nanjing Medical University (Nanjing, China). Patient records/information was anonymized and de-identified prior to analysis, and the methods were carried out in accordance with the approved guidelines.

### Statistical analysis

The association between gender (male or female) and clinicopathological parameters was analyzed by the χ^2^ test. Continuous variables were analyzed using the Student’s *t* test. Survival curves were generated based on Kaplan–Meier estimates, and differences between the curves were analyzed by the log-rank test. Multivariate Cox regression models were generated with hazard ratio (HR) and 95% confidence interval (CI) to analyze risk factors for survival. Statistical analyses were performed using SPSS version 17 for Windows (SPSS Inc., Chicago, IL, U.S.A.). Results were considered statistically significant for a two-tailed *P* value < 0.05.

### Availability of data and materials

The datasets generated and/or analyzed during the present study are available in the SEER dataset repository. https://seer.cancer.gov/.

## Results

### Patient characteristics

We identified 6586 eligible patients with GBM in the SEER database during the 8-year study period (between 2000 and 2008). A total of 4049 (61.5%) were men, and 2537 (38.5%) were women. The median follow-up period was 17 months. The median follow-up period was 17 months in the male group and 19 months in the female group. Patient demographics and pathologic features are summarized in Table 1.

**Table 1:** Characteristics of patients from SEER Database by gender

	Number of patients (%)			
Characteristic	Total	Male	Female	
	*n*=6586	*n*=4049	*n*=2537	*P* value
Media follow up (mo)	17(5–20)	17(5–19)	19(5–22)	
(IQR)				
Years of diagnosis				0.533
2000–2004	3469(52.7)	2145(53.0)	1324(52.2)	
2005–2008	3117(47.3)	1904(47.0)	1213(47.8)	
Age				0.296
<40	581(8.8)	358(8.8)	223(8.8)	
41–60	3635(55.2)	2263 (55.9)	1372(54.1)	
>60	2370(36.0)	1428(35.3)	942(37.1)	
Race				*P*<0.001
Caucasian	5425(82.4)	3361(83.0)	878(34.6)	
African American	914(13.9)	384(9.5)	312(12.3)	
Others*	2282(34.6)	1712(42.3)	209(8.2)	
Primary site				0.870
Cerebrum	235(3.6)	144(3.6)	91(3.6)	
Frontal lobe	1663(25.3)	1028(25.4)	635(25.0)	
Temporal lobe	1556(23.6)	957(23.6)	599(23.6)	
Parietal lobe	1097(16.7)	661(16.3)	436(17.2)	
Occipital lobe	274(4.2)	158(3.9)	116(4.6)	
Ventricle, NOS	27(0.4)	15(0.4)	12(0.5)	
Cerebellum, NOS	36(0.5)	22(0.5)	14(0.6)	
Brain stem	28(0.5)	19(0.5)	9(0.4)	
Overlapping lesion of brain	1158(17.6)	719(17.8)	439(17.3)	
Brain, NOS	512(7.8)	326(8.1)	186(7.3)	
Pathological grading				0.144
High/Moderate	25(0.4)	20 (0.5)	5 (0.2)	
Poor/UD	2655(40.3)	1640(40.5)	1015(40.0)	
Unknown	3906(59.3)	2389(59.0)	1517(59.8)	
Stage				0.127
Localized	5067(76.9)	3152 (77.8)	1915(75.5)	
Regional	1172(17.8)	697(17.2)	475 (18.7)	
Distant	70(1.1)	38(0.9)	32(1.3)	
Unstaged	277(4.2)	162(4.0)	115(4.5)	
Tumor size				0.015
<3 cm	797 (12.1)	463 (11.4)	334 (13.2)	
3–5 cm	2498(37.9)	1511(37.3)	987 (38.9)	
>5 cm	1666(25.3)	1071(26.5)	595(23.5)	
Not stated	1625(24.7)	1004(24.8)	621(24.5)	

Abbreviation: NOS, not otherwise specified.

* including other (American Indian/AK Native, Asian/Pacific Islander) and unknowns.

### Clinicopathological differences between the groups

As illustrated in Table 1, there were significant differences observed between the two groups, including race (more frequent in Caucasian, 82.4%; *P*<0.001) and tumor size (more 3–5 cm, 37.9%; *P*=0.015). Whereas, no differences were observed in years of diagnosis, age, primary site, pathological grading, and stage between the two groups.

### Impact of gender on survival outcomes

**Figure 1:**
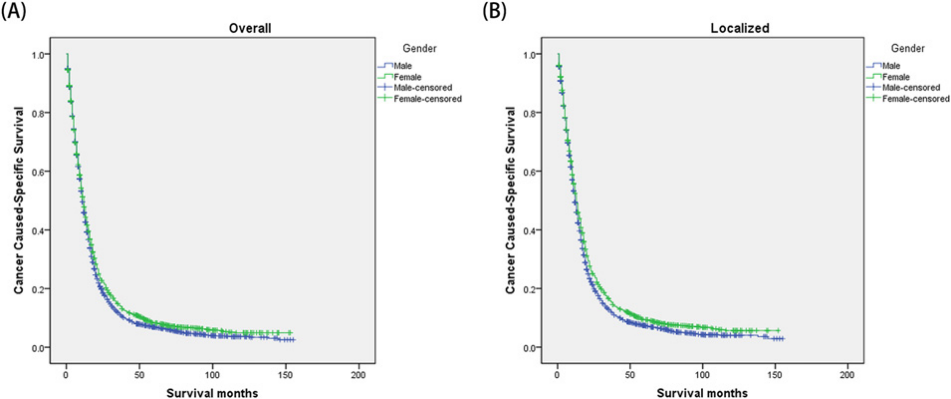
Kaplan–Meier estimates of glioblastoma cancer caused-specific survival in different gender groups (**A**) The overall group; male versus female: χ2 = 9.616, *P*=0.002. (**B**) The localized stage group; male versus female: χ2 = 12.959, *P*<0.001.

The univariate log-rank test showed that the 1-, 3- and 5-year CSS were 45.9%, 11.4% and 6.8% in male group, 47.9%, 14.3% and 8.3% in female group (*P*=0.002) (Figure 1). Moreover, an early year of diagnosis (2000–2004), age more than 60 years, African American race, brain stem tumor, poor/undifferentiated tumor grade (*P*=0.014), higher stage, and larger tumor size (*P*<0.001) were regarded as significant risk factors by univariate analysis (Table 2). Multivariate analysis with Cox regression was performed, and the following seven factors were found to be independent prognostic factors (Table 3), including year of diagnosis (2005–2008: HR, 0.783; 95% CI, 0.743–0.826), gender (female: HR, 0.906; 95% CI, 0.859–0.954), age (41–60 years: HR, 2.036; 95% CI, 1.840–2.254; >60 years: HR, 3.033; 95% CI, 2.729–3.371), race (African American: HR, 1.025; 95% CI, 0.908–1.158), primary site (frontal lobe: HR, 0.996; 95% CI, 0.861–1.151; temporal lobe: HR, 1.018; 95% CI, 0.880–1.178; parietal lobe: HR, 0.953; 95% CI, 0.821–1.107; occipital lobe: HR, 1.025; 95% CI, 0.853–1.232; ventricle, NOS: HR, 1.268; 95% CI, 0.848–1.898; cerebellum, NOS: HR, 1.044; 95% CI, 0.719–1.516; brain stem: HR, 1.518; 95% CI, 1.023–2.254; overlapping lesion of brain: HR, 0.983; 95% CI, 0.847–1.141; brain, NOS: HR, 0.787; 95% CI, 0.667–0.929); pathological grading (poor/undifferentiated: HR, 1.418; 95% CI, 0.912–2.205), stage (regional: HR, 1.568; 95% CI, 1.465–1.678; distant: HR, 1.580; 95% CI, 1.238–2.017), tumor size (3–5 cm: HR, 1.029; 95% CI, 0.946–1.119; >5 cm: HR 1.145; 95% CI, 1.046–1.253).

**Table 2:** Univariate survival analyses of GBM patients according to various clinicopathological variables

Variable	*N*	1-year CSS (%)	3-year CSS (%)	5-year CSS (%)	Log rank χ2 test	*P*
Years of diagnosis					61.795	*P*<0.001
2000–2004	3469	41.7%	10.2%	6.5%		
2005–2008	3117	52.1%	15.0%	8.3%		
Gender					9.616	0.002
Male	4049	45.9%	11.4%	6.8%		
Female	2537	47.9%	14.3%	8.3%		
Age					477.901	*P*<0.001
<40	581	74.3%	35.0%	26.1%		
41–60	3635	50.5%	12.3%	6.9%		
>60	2370	33.7%	7.2%	3.5%		
Race					29.078	*P*<0.001
Caucasian	5425	46.2%	11.6%	7.0%		
African American	914	45.8%	16.8%	9.9%		
Others*	2282	55.8%	24.3%	13.9%		
Primary site					51.553	*P*<0.001
Cerebrum	235	49.5%	11.2%	6.2%		
Frontal lobe	1663	47.6%	11.1%	7.1%		
Temporal lobe	1556	44.2%	10.8	5.5		
Parietal lobe	1097	45.3%	14.3%	7.9%		
Occipital lobe	274	42.3%	11.8%	4.7%		
Ventricle, NOS	27	29.6%	3.7%	NI		
Cerebellum, NOS	36	53.5%	11.9	5.9		
Brain stem	28	28.6%	3.6%	NI		
Overlapping lesion of brain	1158	46.3%	13.3%	7.9%		
Brain, NOS	512	56.9%	18.3%	13.9%		
Pathological grading					8.529	0.014
High/Moderate	25	68.0%	32.0%	22.4%		
Poor/UD	2655	46.8%	12.0%	7.2%		
Unknown	3906	46.4%	12.7%	7.4%		
Stage					159.412	*P*<0.001
Localized	5067	50.5%	13.7%	8.1%		
Regional	1172	32.1%	7.3%	4.8		
Distant	70	30.3%	4.5%	NI		
Unstaged	277	41.4%	13.7%	7.9		
Tumor size					29.108	*P*<0.001
<3 cm	797	53.2	14.0	7.5		
3–5 cm	2498	49.7	13.1	7.6		
>5 cm	1666	43.4	12.4	7.5		
Not stated	1625	42.0	10.8	7.1		

Abbreviation: NI, not included.

* including other (American Indian/AK Native, Asian/Pacific Islander) and unknowns.

**Table 3:** Multivariate Cox model analyses of prognostic factors of GBM

Variable	Hazard ratio	95%CI	*P*
Years of diagnosis			*P*<0.001
2000–2004	1	Reference	
2005–2008	0.783	0.743–0.826	
Gender			
Male	1	Reference	*P*<0.001
Female	0.906	0.859–0.954	
Age			*P*<0.001
<40	1	Reference	
41–60	2.036	1.840–2.254	
>60	3.033	2.729–3.371	
Race			*P*<0.001
Caucasian	1	Reference	
African American	1.025	0.908–1.158	
Others*	0.750	0.663–0.848	
Primary site			*P*<0.001
Cerebrum	1	Reference	
Frontal lobe	0.996	0.861–1.151	
Temporal lobe	1.018	0.880–1.178	
Parietal lobe	0.953	0.821–1.107	
Occipital lobe	1.025	0.853–1.232	
Ventricle, NOS	1.268	0.848–1.898	
Cerebellum, NOS	1.044	0.719–1.516	
Brain stem	1.518	1.023–2.254	
Overlapping lesion of brain	0.983	0.847–1.141	
Brain, NOS	0.787	0.667–0.929	
Pathological grading			0.178
High/Moderate	1	Reference	
Poor/UD	1.418	0.912–2.205	
Unknown	1.454	0.935–2.259	
Stage			*P*<0.001
Localized	1	Reference	
Regional	1.568	1.465–1.678	
Distant	1.580	1.238–2.017	
Unstaged	0.988	0.865–1.129	
Tumor size			*P*<0.001
<3 cm	1	Reference	
3–5 cm	1.029	0.946–1.119	
>5 cm	1.145	1.046–1.253	
Not stated	1.158	1.056–1.269	

* including other (American Indian/AK Native, Asian/Pacific Islander) and unknowns.

### Stratified analysis of gender effect on CSS rates

**Figure 2:**
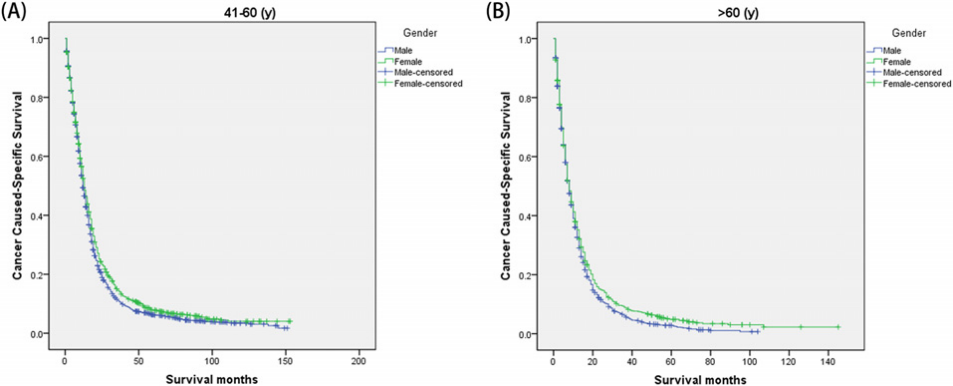
Subgroup analysis for evaluating the effect of gender for glioblastoma patients according to different age (**A**) 41–60 years: χ2 = 8.389, *P*=0.004; (**B**) >60 years: χ2 = 6.233, *P*=0.013.

We then further analyzed the effect of gender on CSS rates in each stage (Figure 1). The univariate analysis of gender on CSS showed that female had increased 1-, 3-, and 5-year CSS in localized stage (*P*<0.001), but not in regional (*P*=0.619) and distant stage (*P*=0.259). And gender was validated as an independent predictor of survival in multivariate Cox regression in the localized stages (*P*<0.001) (Figure 1) (Table 4). Furthermore, we made further stratified analysis of survival rates and hazard by age (Figure 2). Male always had the lowest CSS rate in 41–60 years and >60 years group, which were consistent with above results (Table 5).

**Table 4:** Univariate and multivariate analyses for evaluating gender influencing CSS in GBM based on different cancer stage

	Univariate analysis					Multivariate analysis	
Variable	1-year CSS (%)	3-year CSS (%)	5-year CSS (%)	Log rank χ2 test	*P*	HR (95%CI)	*P*
Localized							
Gender				12.959	*P*<0.001		*P*<0.001
Male	49.3	12.4	7.2			Reference	
Female	52.4	15.8	9.3			0.898(0.845–0.954)	
Regional							
Gender				0.247	0.619		
Male	31.7	6.3	5.1%				
Female	32.7	8.6	4.4%				
Distant							
Gender				1.273	0.259		
Male	39.4	5.6	NI				
Female	16.4	3.3	NI				

**Table 5:** Univariate and multivariate analyses for evaluating gender influencing CSS in GBM based on different age

	Univariate analysis					Multivariate analysis	
Variable	1-year CSS (%)	3-year CSS (%)	5-year CSS (%)	Log rank χ2 test	*P*	HR (95%CI)	*P*
<40							
Gender				0.072	0.788		NI
Male	75.9	34.6	26.1%				
Female	71.9	35.6	24.9%				
41–60							
Gender				8.389	0.004		0.003
Male	49.3	11.0	6.1%			Reference	
Female	52.5	14.4	7.8%			0.899(0.837–0.965)	*P*<0.001
>60							
Gender				6.233	0.013		0.029
Male	32.6	6.0	2.4%			Reference	
Female	35.2	8.9	4.9%			0.908(0.832–0.990)	

NI: not included in multivariate survival analysis.

*P* values were adjusted for years of diagnosis, age, race, pathological grading, stage and tumor size as covariates between the two groups.

## Discussion

GBM accounts for 17% of intracranial tumors and be considered as the most common brain tumor in adults [13]. Despite surgical resection followed by adjuvant radiotherapy and chemotherapy has been applied, prognosis remains poor and long-term survival is rare [14]. Thus, further understanding and improvements in GBM prognosis may affect the choice of salvage therapy and follow-up strategies.

The higher percentage of GBM in men compared with women has been reported in some literature, with a mean male/female ratio ranging from 1.0 to 1.9 [15–17]. However, to the best of our knowledge, there is limited information regarding the impact of sex on survival in patients with GBM. Our study revealed a correlation between female sex and improved CSS and OS in patients with GBM. This survival discrepancy still existed after stratified analysis. Interestingly, female patients have an equivalent percentage in poor/undifferentiation grade (40.0% versus 40.5%) and more than 3 cm tumor size (63.8% versus 62.4%) when compared with male patients. In addition, even after adjusting confounding factors, gender remained to serve as an independent prognostic predictor.

Sex disparities in cancer mortality arise from the sex differences have been analyzed widely. However, the evidence regarding the influence of reproductive factors and hormones on GBM has not been well verified. Epidemiological studies provided very limited evidence regarding the impact of sex on survival in patients with GBM [18–21]. Some studies have reported that female have longer survival than male [22,23]. Barone et al. [24] demonstrated that estrogen increased survival in an orthotopic model of glioblastoma, and estradiol-based study may be beneficial in treating GBM. Li et al. [25]observed high frequency of estrogen receptor methylation GBMs, indicating that estrogen protect patients from GBM. Moreover, Yu et al. [26] found that androgen receptor signaling could promote tumorigenesis of GBM in adult men by inhibiting TGF-β (transforming growth factor β) receptor signaling. The findings of our study suggest that estrogen may protect against GBM genesis and promote a more favorable biology once GBM develops.

Univariate analysis showed that female had a better 1-, 3-, and 5-year CSS compared with male patients, but this failed to reach statistical significance in multivariable Cox regression models of regional and distant stages. A total of 4049 male GBM patients and 2537 females were included in our study, the largest sample size up to now. Due to the protective role of estrogen in the female groups, these patients exhibited better survival. The survival disadvantage in women aged more than 60 years may reflect the lasting effect of estrogen on the biology of GBM. In addition to the impact of sex on survival, we explored potential interactions between sex and age. Male patients were at an increased risk of cancer mortality in contrast with female patients with different age subgroups after adjusted for confounding factors. When comparing with male patients, female patients always had the worse CSS in regional and distant subgroups.

Although the present study is based on a large population, there are still limitations. First, its retrospective nature may affect the analysis. Second, several important pieces of information regarding GBM predisposing factors were not included in the SEER database. Moreover, current classification of tumors of the CNS does not include the term glioblastoma multiforme, thus we cannot adjust the nomenclature according to the newest criteria. Besides, information on menopausal status or use of hormone therapy was not included in the SEER database, thus limit our ability to reach definitive conclusions in this regard. Despite these limitations, our large population-based study may render our conclusions more convincing.

## Conclusions

The results of the present study demonstrate that sex influences survival among patients with GBM. Compared with male patients, female patients with GBM have a higher CSS after surgery. Future studies are warranted to validate these confounding factors and present unique opportunities for novel therapeutics.

## Abbreviations

CSS: cancer-specific survival
GBM: glioblastoma multiforme
HR: hazard ratio
SEER: Surveillance, Epidemiology, and End-Results
